# Histone Modification of Osteogenesis Related Genes Triggered by Substrate Topography Promotes Human Mesenchymal Stem Cell Differentiation

**DOI:** 10.1021/acsami.3c01481

**Published:** 2023-06-13

**Authors:** Xun Xu, Weiwei Wang, Jie Zou, Karl Kratz, Zijun Deng, Andreas Lendlein, Nan Ma

**Affiliations:** † Institute of Active Polymers and Berlin-Brandenburg Centre for Regenerative Therapies, Helmholtz-Zentrum Hereon, 14513 Teltow, Germany; ‡ Institute of Chemistry and Biochemistry, Free University of Berlin, 14195 Berlin, Germany; § Helmholtz Virtual Institute − Multifunctional Biomaterials for Medicine, 14513 Teltow and Berlin, Germany; ∥ Institute of Chemistry, University of Potsdam, 14469 Potsdam, Germany

**Keywords:** topographical cues, hBMSCs, cell contractility, histone modification, osteogenic differentiation

## Abstract

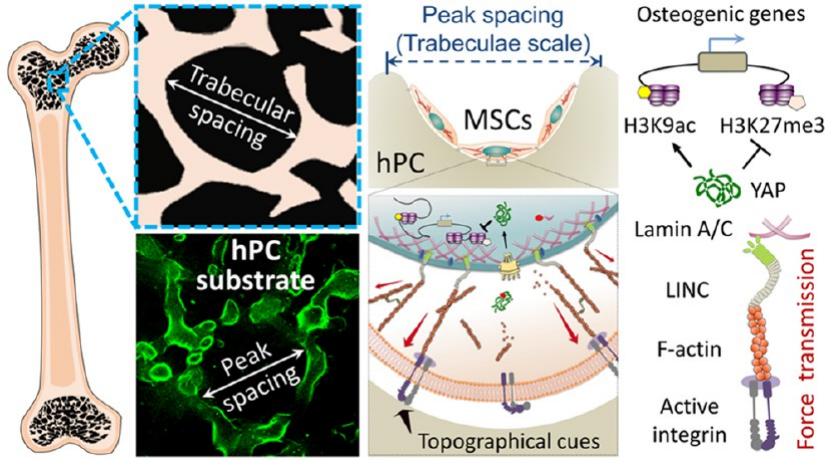

The clinical success of orthopedic implants is closely related to their integration in the bone tissue promoted by rough device surfaces. The biological response of precursor cells to their artificial microenvironments plays a critical role in this process. In this study, we elucidated the relation between cell instructivity and surface microstructure of polycarbonate (PC)-based model substrates. The rough surface structure (hPC) with an average peak spacing (Sm) similar to the trabecular spacing of trabecular bone improved osteogenic differentiation of human bone marrow mesenchymal stem cells (hBMSCs), as compared to the smooth surface (sPC) and the surface with a moderate Sm value (mPC). The hPC substrate promoted the cell adhesion and assembling of F-actin and enhanced cell contractile force by upregulating phosphorylated myosin light chain (pMLC) expression. The increased cell contractile force led to YAP nuclear translocation and the elongation of cell nuclei, presenting higher levels of active form of Lamin A/C. The nuclear deformation alternated the histone modification profile, particularly the decrease of H3K27me3 and increase of H3K9ac on the promoter region of osteogenesis related genes (*ALPL*, *RUNX2*, and *OCN*). Mechanism study using inhibitors and siRNAs elucidated the role of YAP, integrin, F-actin, myosin, and nuclear membrane proteins in such a regulatory process of surface topography on stem cell fate. These mechanistical insights on the epigenetic level give a new perspective in understanding of the interaction of substrate and stem cells as well as provide valuable criteria for designing bioinstructive orthopedic implants.

## Introduction

In orthopedic surgery, the microstructure of the implant surface is a crucial factor determining the long-term stability of osseointegrated implants. Currently, the usage of implant with a rough surface has been proved to be effective for improving the stable integration between the surgically placed implants and bone tissue.^1−3^ Compared to a smooth surface, the implants with microroughness structure can provide more space for cell adhesion and increase the bone anchorage, thereby reinforcing the biomechanical interlocking between bone and implants.^4,5^ The implants with different microroughness levels on their surfaces have been applied in clinical treatment and preclinical studies. Some reports suggested that a surface with higher roughness ensured a higher surface area and in this way a relatively larger bone to implant contact area, which enhanced osteoconductivity and osteogenesis, and thereby improved osseointegration.^6^ However, in other studies, the more pronounced bone responses were obtained on surfaces with moderate roughness (Ra = 1–2 μm) than on other roughness levels;^7^ while the surface with the roughness values Ra ≈ 4.5 μm favored osseointegration compared to those with lower roughness (Ra < 2 μm).^8,9^ Conclusively, it is unclear which roughness level optimally favors osseointegration. A clear criterion is required for designing implant surface with microroughness.

Mesenchymal stem cells (MSCs) are well-known for their multipotent differentiation potential and have been applied for bone tissue regeneration. Their cellular functions could be regulated by topographical cues of the cell culture substrates.^10−12^ Such cues can be sensed and transferred to biological signals by MSCs, resulting in cytoskeletal remodeling and change of cell contractility.^13^ The cell contractile force can be transmitted to the nucleus, inducing nuclear deformation and influencing the conformation and phosphorylation of nuclear proteins.^14,15^ For example, the mechanosensitive proteins Lamin A/C, which are located on the inner nuclear membrane and internal nuclear scaffold, respond to the contractile force via the turnover of phosphorylated Lamin A/C (pLamin A/C) and Lamin A/C.^16,17^ The mechanical force can lead to an increase of nuclear membrane tension and improves the permeability of nuclear pore complexes (NPC),^18^ which promotes nuclear entry of the mechanosensitive transcription coactivator yes-associated protein (YAP).^19^ After transmission to the nucleus, mechanical force may exert an effect on histone acetylation/methylation and the chromatin dynamics.^20−23^ A rapid increase of gene transcription was observed after the cells were exposed to a mechanical force for a short time period.^24^ Long-term application of a force (12 h) on MSCs could lead to an enhancement of trimethylation of histone H3K27 (H3K27me3), which resulted in transcription repression and affected the stem cell lineages.^25^ The internal force generated by activated actomyosin was able to regulate the methylation and acetylation of histone H3 and then modulate the expression of specific genes.^26^ The histone acetylation and methylation patterns on the promoters of specific genes controlled the activation and silencing of transcription.^27−30^ H3K27me3 has been shown to be an epigenetic control point of adipogenesis of stem cells,^31^ while the acylation of H3K9 (H3K9ac) played a key role in regulating the proliferation and osteogenic differentiation of stem cells.^32^

**Figure 1:**
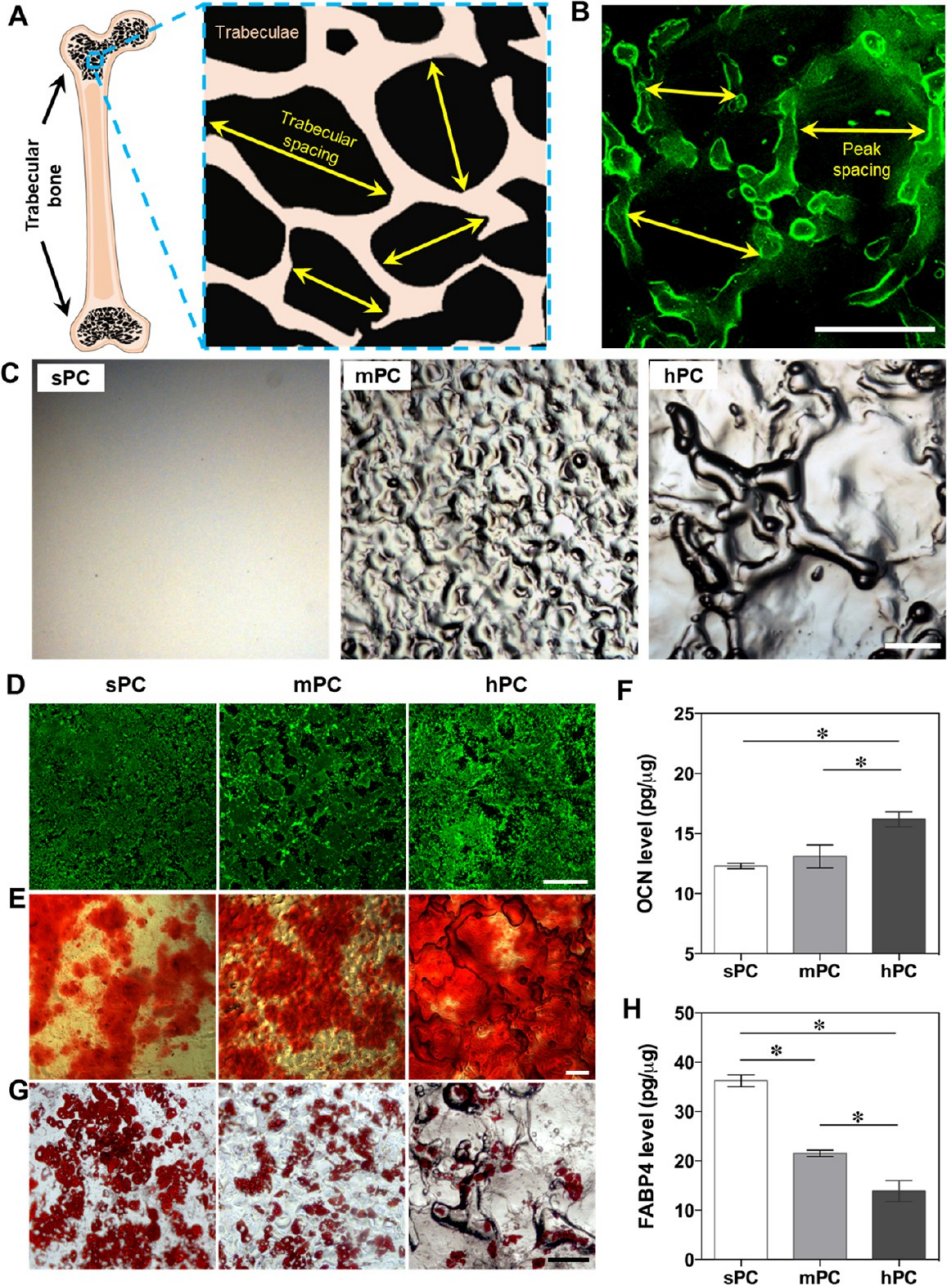
Regulation of hBMSC differentiation using 2.5D substrate with bioinspired surface topographical cues mimicking the structure of trabecular bone. (A) Schematic representation of the structure of human trabecular bone. (B–C) PC-based substrates with different levels of roughness were used as model materials. Confocal laser scanning microscopic image (B) demonstrated that the average peak spacing (Sm) of hPC substrate is comparable to the pore size of trabecular bone. To visualize the microstructure, the substrate was coated with fibronectin and stained with fluorochrome conjugated fibronectin antibody. The green fluorescence indicated the fibronectin on the peak area of the substrate. Phase contrast microscopic images (C) showed the microstructure of all PC substrates (scale bar = 100 μm). (D–H) hBMSC differentiation on different substrates. The cells were first cultured in GM for 4 days, then the medium was replaced by MM and the cells were further cultured for 21 days. Staining was performed to visualize the hydroxyapatite (D), calcium deposits (E), and lipid droplets (G) (scale bar = 200 μm). OCN (F) and FABP-4 (H) protein levels of hBMSCs were normalized by the amount of total protein (*n* = 4; * *p* < 0.05).

MSCs lineage commitment was strongly influenced by the microarchitecture of a culture substrate.^23,33,34^ Designing an appropriate surface topography of orthopedic implants would be an effective strategy for improving osseointegration via guiding MSC osteogenic differentiation to form new bone tissue. Only a few mechanisms of biological effects caused by surface microstructure have been identified to date. Therefore, in order to define the design criteria, the mechanism about the transmission of the topographical signal from cell membrane to the nucleus as well as the consequently triggered intranuclear events needs to be considered. The epigenetic regulation of cells by surface structure during the osteogenic process provides a guide to engineer the implant surface with topographical cues for improving the therapeutic efficacy. Bone marrow, particularly the red bone marrow, is a major cell source of MSCs typically filling the multiporous structure of trabecular bone in adult tissue.^35^ Trabecular bone, as the native microenvironment of human bone marrow MSCs (hBMSCs), has a spongy-like morphology, which is highly heterogeneous and anisotropic (Figure 1A). Although the pore size (trabecular spacing) of trabecular bone is dependent on the age and position of bone and varies in different individuals,^36,37^ the largest frequency of the pore sizes is in the range from 200 to 600 μm.^38,39^ Compared to cortical bone, trabecular bone has a larger surface area, with marrow and blood vessels filled between the trabeculae. Trabecular bone has a much faster remodeling process than cortical bone,^40^ suggesting the interior microenvironment may favor bone formation. Inspired by the microstructure of human trabecular bone, we hypothesized that a 2.5D rough surface substrate with an average peak spacing (Sm) comparable to the pore size of trabecular bone might improve the hBMSC osteogenic differentiation by providing a structural microenvironment similar to trabecular bone. Here, polycarbonate (PC) was selected for its high biocompatibility, transparency, and elastic modulus that is comparable to native human trabeculae.^41,42^ The PC-based inserts with different roughness levels on the bottom were fabricated as the substrate materials. Inserts with a smooth surface (sPC) were used as a control. A surface (mPC) with a moderate Sm value (160 ± 8 μm) comparable to the dimension of single MSC (∼100 μm in adhesion), as well as a surface (hPC) with a high Sm value (280 ± 30 μm) similar to the pore size of trabecular bone (Figure 1B) were applied as the 2.5D substrates. The transmission of the topographical signal from the cell–material interface into cytoplasm and the cell nuclei was investigated. The osteogenic differentiation of hBMSCs on the substrates was assessed. Intranuclearly, the biological effect, including the YAP activation and histone modification, triggered by the topographical signals was studied, and the underlying mechanism was elucidated using small molecule inhibitors and siRNAs.

## Results and Discussion

### Characterization of Substrate Topography

The inserts were fabricated from PC with different microstructures on the bottom (Figure 1C), which were characterized with optical profilometry and AFM (Figure S1 and Table S1). The mPC substrate has a Sm value (160.3 ± 8.2 μm) comparable to the dimension of a single hBMSC, and the hPC has a Sm value (279.3 ± 32.3 μm) similar to the pore size of trabecular bone. At the nanoscale, the roughness Ra was less than 5 nm for all substrates.

**Figure 2:**
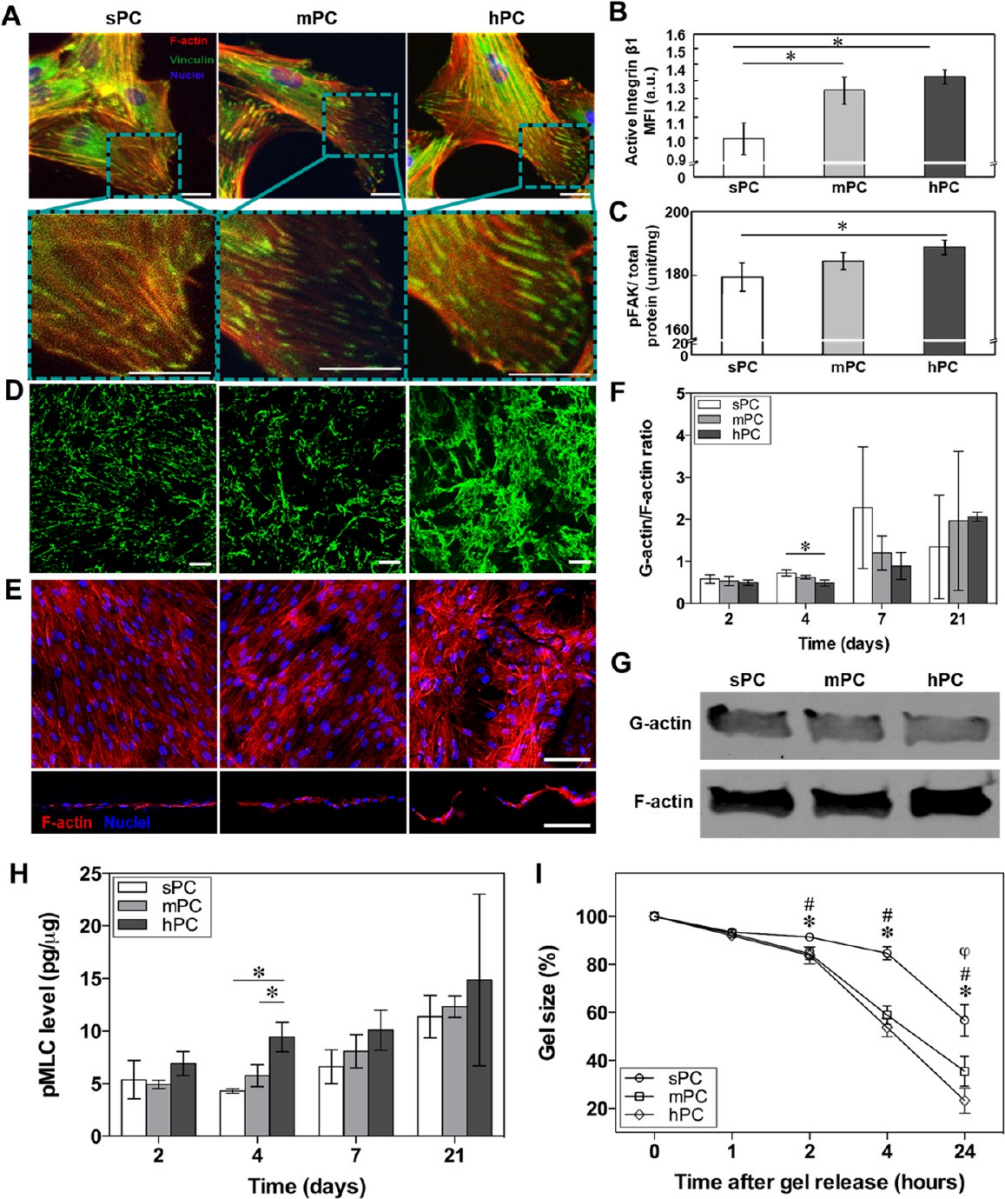
hPC surface topographical cues promote hBMSC adhesion, actin polymerization, and contractility. (A–D) Analysis of focal adhesion of hBMSCs cultured on different substrates in GM for 4 days. (A) Representative fluorescence images shown the vinculin and F-actin staining in hBMSCs (green: vinculin; red: F-actin; blue: nuclei; scale bar = 10 μm). (B,C) Quantification of active integrin β1 (*n* = 4; * *p* < 0.05) and phosphorylation (Y397) levels of FAK (*n* = 3; * *p* < 0.05) in hBMSCs. (D) Representative staining image of fibronectin secreted by hBMSCs (scale bar = 100 μm). (E) Representative top view (upper) and cross-sectional view (lower) of immunofluorescence staining images of hBMSCs cultured on different substrates in GM for 4 days (red: F-actin; blue: nuclei; scale bar = 100 μm). (F) Quantitative analysis of G-actin/F-actin ratio of hBMSCs cultured in GM (*n* = 3; * *p* < 0.05). (G) Representative Western blot images of G-actin and F-actin of cells cultured in GM for 4 days. (H) The pMLC expression level of hBMSCs cultured on different substrates (*n* = 3; * *p* < 0.05). (I) Contractility of cells cultured in GM for 4 days was assessed using the cell mixed collage gels (*n* = 4; * *p* < 0.05, sPC vs mPC; # *p* < 0.05, sPC vs hPC; φ *p* < 0.05, mPC vs hPC).

### Substrate Microstructure Regulates hBMSC Differentiation

The osteogenic and adipogenic differentiation of hBMSCs was examined to verify whether the topographical cues could regulate hBMSC differentiation. Since cell–cell interaction could influence MSC differentiation,^43^ the expression level of N-cadherin was determined. There was no significant difference of N-cadherin expression levels among the tested groups (Figure S2A,B), suggesting that the cells on the different substrates would experience similar cell–cell interactions. The influence of substrate on hBMSC differentiation was first evaluated by culturing the cells in pure induction medium (osteogenic or adipogenic induction medium). Alizarin Red S and FABP-4 staining showed that hPC promoted hBMSC osteogenesis, while sPC was favorable for hBMSC adipogenesis (Figure S3). Further, we examined the cell differentiation in mixed induction medium (MM), which contains both osteogenic and adipogenic induction components to better mimic the complex and dynamic environment that MSCs experience *in vivo*. A similar effect of substrate microstructures on hBMSC differentiation was found when the cells were cultured in MM. After 21 days of culture, the highest levels of hydroxyapatite (Figure 1D), calcium deposits (Figure 1E), and OCN expression (Figure 1F) were observed in the hPC group. The cells on hPC showed a lower level of adipogenic differentiation than on sPC and mPC, as evidenced by fewer lipid droplets (Figure 1G) and the significantly downregulated expression of adipogenic differentiation marker FABP-4 (Figure 1H). These results suggested that hPC promoted hBMSC osteogenic differentiation and inhibited the adipogenic differentiation, regardless of the induction conditions used.

### Substrate Microstructure Regulates Cell Adhesion, Cytoskeleton Formation, and Cell Contractility

The process of mechanotransduction and differentiation of stem cells is highly dependent on the cell adhesion to the substrate.^44,45^ Our previous study has demonstrated that the microstructure on the hPC surface enhanced the expression of total integrin α2, activation of integrin α3, and secretion of the ECM component Laminin-5 in hBMSCs, in comparison to sPC and mPC substrates.^46^ In this study, we further examined the focal adhesion complex, integrin β1 subunit activation, and FAK phosphorylation, all of which play a major role in promoting MSC osteogenic differentiation and osteoblast maturation.^47−49^ We observed that hBMSCs exhibited a greater number of focal adhesions on hPC substrate, as evidenced by vinculin staining (Figure 2A). Compared to sPC, hBMSCs cultured on hPC showed significantly elevated levels of activated integrin β1 and FAK phosphorylation (Figure 2B,C). The ECM component fibronectin has been proven to regulate fracture healing and promote stem cell differentiation along skeletal lineages while suppressing adipogenic differentiation.^50,51^ We found that hBMSCs cultured on hPC substrates secreted and deposited a higher level of fibronectin than those cultured on sPC and mPC (Figure 2D). Taken together, our findings suggested that the microstructure of hPC can promote integrin activation and its downstream FAK signaling, as well as regulate the secretion and distribution of ECM proteins and their interaction with cells.

The actin cytoskeleton is a dynamic filament system, which constantly reorganizes itself by polymerization and depolymerization cycles to regulate cell adhesion, shape, and migration to adapt to the environment. F-actin as a component of the cytoskeleton was regulated by the cell’s local environment and was involved in various cell signaling pathways. The F-actin amount and organization directly modulated cell stiffness^52^ and MSC osteogenic differentiation.^53^ The bone formation could be enhanced via inhibiting F-actin depolymerization.^54^ Here, the cells cultured on hPC presented a stronger F-actin signal than the cells cultured on sPC and mPC. In contrast to the cells on sPC and mPC with F-actin in a highly aligned orientation, the cells on hPC presented randomly orientated F-actin (Figure 2E upper panel). The cross-sectional view images of hBMSCs on hPC indicated that there was a stronger F-actin signal on the slopes than on other positions (Figure 2E lower panel). These results indicated that the surface topographical cues influenced the actin polymerization and cytoskeleton arrangement. The value of G-actin/F-actin ratio presented a decreasing trend with the increase of substrate roughness in the first week. On day 4, a significantly lower G-actin/F-actin ratio was observed on hPC in comparison with sPC substrate (Figure 2F,G). However, an opposite trend was observed on day 21, which might be attributed to the high cell confluence and restricted migration.^55,56^

Myosin II is the major motor protein usually in association with F-actin, playing a critical role for generating the intracellular contractile force to guide cell spreading, migration, division, as well as differentiation.^57,58^ The activation of myosin II is regulated by phosphorylation of myosin light chains. The level of phosphorylated myosin light chain (pMLC) showed an increasing trend with the increase of substrate surface roughness (Figure 2H). Given the role of F-actin and pMLC for mediating contractile force, we harvested the cells cultured in GM for 4 days and evaluated the cell contractility using the cell–collagen mixed gels method. After 24 h, the highest shrinkage was observed in the gel containing the cells derived from hPC (Figure S4). Compared to the initial gel (0 h), the size of the gel containing cells from hPC decreased to 23 ± 5% after 24 h, which was significantly lower than the gels with cells from sPC (57 ± 7%) and mPC (35 ± 6%) (Figure 2I). These results indicated that the hPC substrate could effectively increase the contractility of hBMSCs.

### hPC Substrate Induces Nuclear Deformation and Enhances Nucleoskeleton

**Figure 3:**
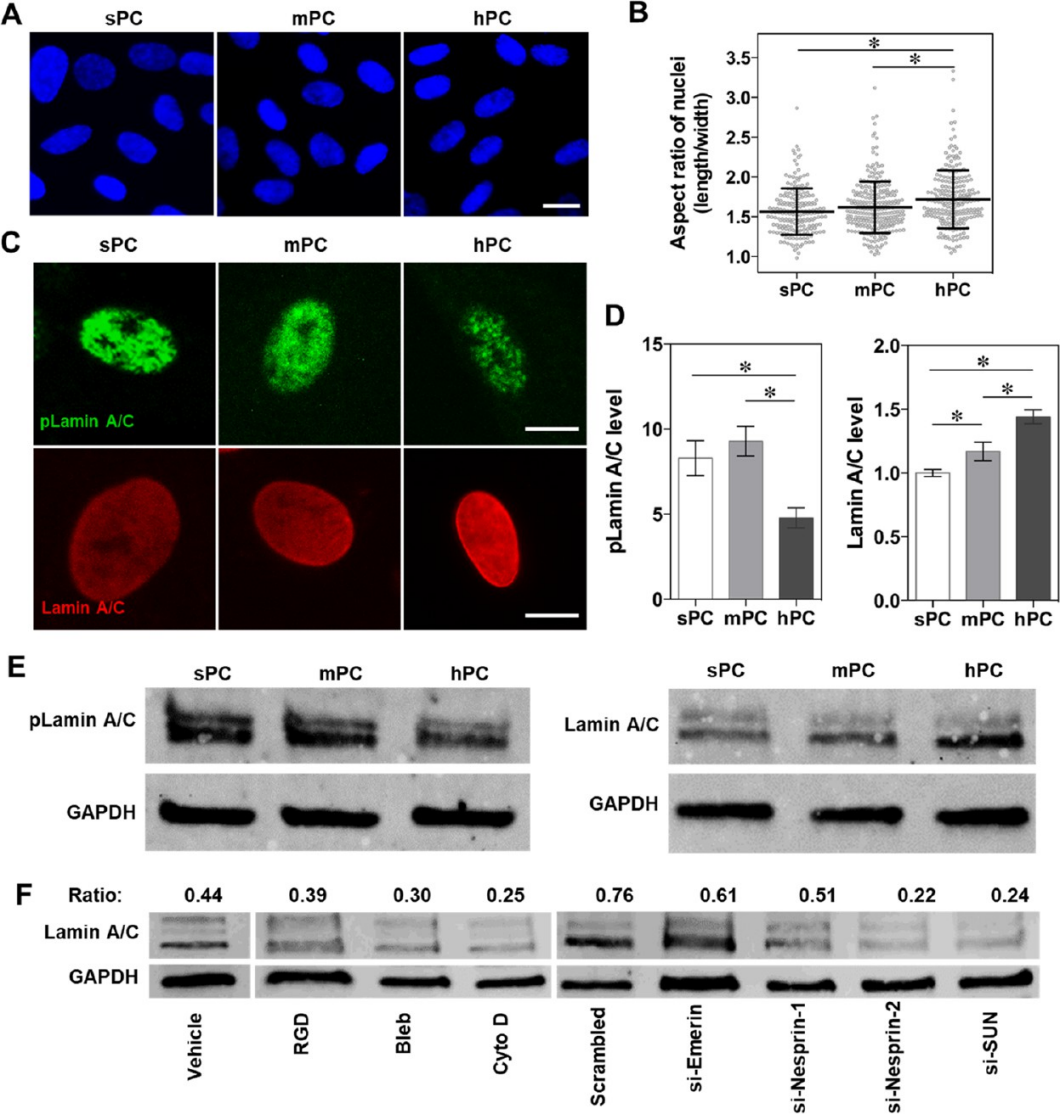
hPC substrate enhances nuclear elongation and Lamin A/C enrichment. The hBMSCs were culture on different substrates in GM for 4 days. (A) Representative cell nuclei staining images of hBMSCs (scale bar = 20 μm). (B) Quantitative analysis of nuclei aspect ratio of cells (*n*_nuclei_ = 195 (sPC), 251 (mPC), and 219 (hPC); * *p* < 0.05). (C) Representative immunofluorescence staining images of nuclear pLamin A/C and Lamin A/C of hBMSCs (scale bar = 10 μm). (D, left panel) Quantification of mean fluorescence intensity (MFI) of pLamin A/C based on the staining images (bars show the standard error of the mean (SEM); *n*_nuclei_ = 35 (sPC), 40 (mPC), and 28 (hPC); * *p* < 0.05). (D, right panel) Lamin A/C expression level of hBMSCs was quantified using flow cytometry, and the MFI was calculated with Flowjo software. The average value of the sPC group was set as 1 (*n* = 5; * *p* < 0.05). (E) Western blot images of pLamin A/C, Lamin A/C, and GAPDH of hBMSCs. (F) Western blot analysis of Lamin A/C. The cells cultured on hPC were treated with inhibitors or transfected with siRNAs. Vehicle and scrambled siRNA groups were served as controls, respectively. The protein amount was analyzed using ImageJ software, and the ratio of Lamin A/C to GAPDH was listed on top.

The cytoskeleton network bridges the cell membrane and the nucleus through the linker of nucleoskeleton and cytoskeleton (LINC) complex.^59−61^ In this way, the mechanical signals could be directly transmitted from the cell/material interface to the nucleus.^62^ Compared to the relaxed nucleus under low intracellular tension, the mechanically stressed nucleus deformed as a response to the high contractile force.^63^ Here, the nuclei of cells cultured on hPC were obviously “slender” than on other substrates (Figure 3A), with a significantly higher nuclei aspect ratio (Figure 3B).

Lamin A/C as major components of the nuclear lamina provide the structural integrity to cell nucleus and mechanical support to nuclear shape.^64^ The stimulation of mechanical force can enhance the dephosphorylation of phosphorylated Lamin A/C (pLamin A/C), inducing conformational changes into Lamin A/C and localization on the inner nuclear membrane.^20^ The expression level of Lamin A/C determines the cell nuclear stiffness. Several studies found that Lamin A/C interacted with double-stranded DNA, transcriptional regulators, and nuclear membrane associated proteins to regulate the expression of genes that are related to MSC differentiation.^64,65^ Here, the cells on hPC expressed the lowest level of pLamin A/C but the highest level of Lamin A/C (Figure 3C,D,E), indicating the mechanical force generated by hPC topography could enhance Lamin A/C on nuclear membrane and inhibit their phosphorylation, which was consistent with the previous report.^66^

Conclusively, these results suggested that the hPC topographical cues could induce nuclear elongation and enhance the Lamin A/C localization on the inner nuclear membrane. This observation might be explained with the following mechanism. Compared to sPC and mPC substrates, the structural topography of hPC effectively influenced the cell distribution. The huge peaks and deep valleys on hPC led to the “rolling” of initially seeded cells (not attached) to the valleys and restricted the later cell migration. As a result, more hBMSCs were found in the valley area especially when seeded at a high cell density, in contrast to the homogeneous cell layer on sPC and mPC substrates (Figure S5). The majority of hBMSCs on hPC would experience the concave microcurvature of the valley area. Considering the promoted F-actin cytoskeleton formation and enhanced pMLC level by concave microcurvature,^67^ the hPC substrate would increase the contractile force of cells in this way and consequently induce nuclear deformation and Lamin A/C localization.

To clarify the mechanism at the molecular level, through which the substrate microstructures modulate the cells, we used the small molecule inhibitors and siRNAs (according to the quantitative analysis, the knockdown efficiency of Lamin A/C was confirmed to be 50% (Figure S6)) to disrupt the functions of components involved in the mechanosensation and mechanotransduction processes. Blockage of surface integrin and inhibition of myosin II activity and actin polymerization with RGD peptide, blebbistatin (Bleb), and cytochalasin D (Cyto D) reduced the Lamin A/C expression (Figure 3F), suggesting the basic functional role of these components for sensing and transducing the mechanical signals to cell nuclei. In addition, Lamin A/C expression was reduced when the cells were transfected with siRNAs to interfere the LINC complex component proteins (Nesprin-1, Nesprin-2, and SUN), which connect the nucleoskeleton to the cytoskeleton and transmit the mechanical force from the cytoplasm to the nucleus. However, interference of the LINC complex protein Emerin did not show any apparent effect on Lamin A/C expression (Figure 3F). One reason might be that Emerin, although functioning as a mechanical sensor protein, does not participate in such a force conduction process due to its lack of direct contact to the cytoskeleton elements.^18^

### hPC Substrate Regulates YAP Nuclear Translocation and Activity

**Figure 4:**
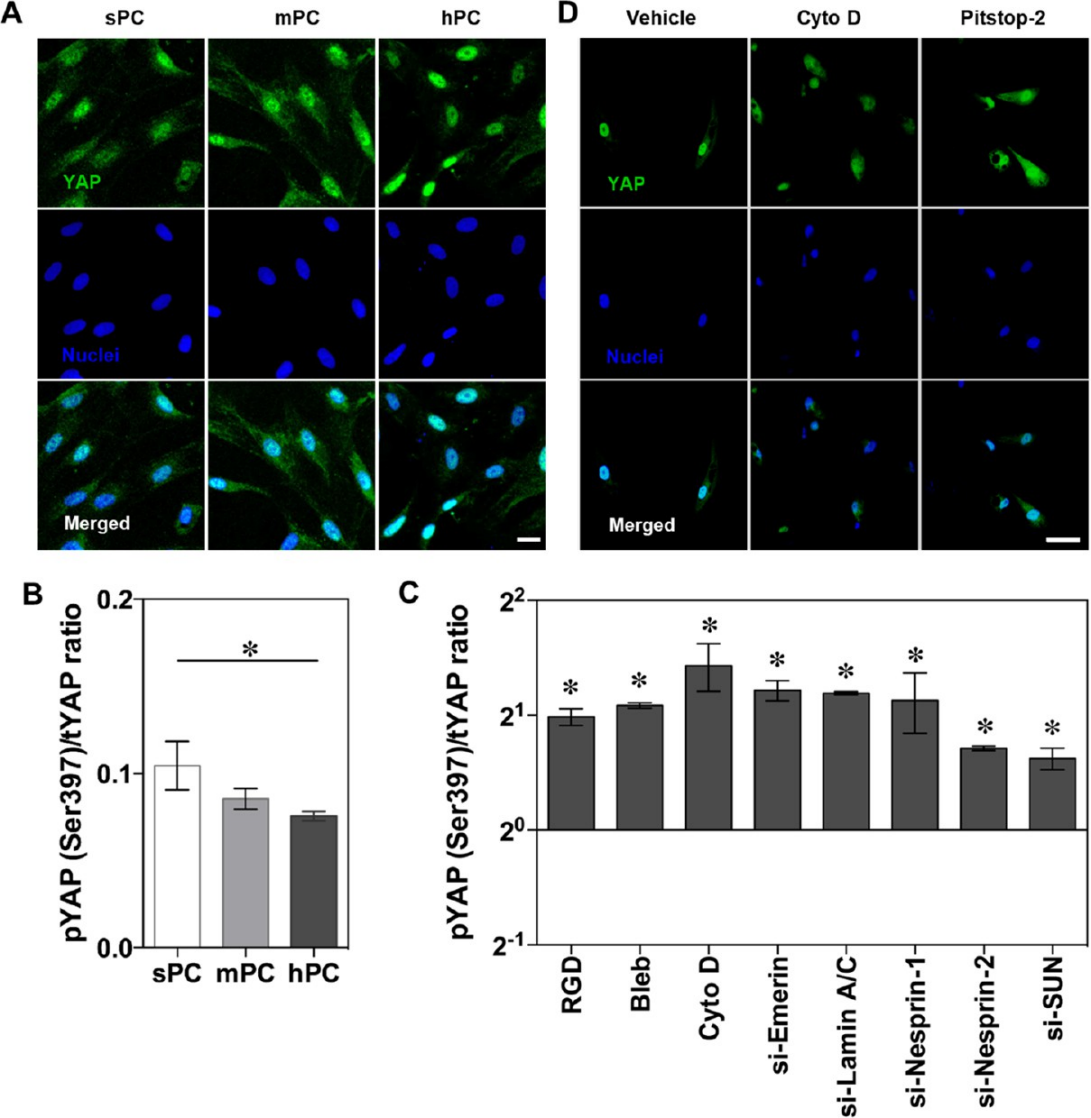
YAP activation and nuclear translocation in response to substrate topographical cues. The hBMSCs were cultured on different substrates in GM for 4 days. (A) Representative immunofluorescence staining images of YAP and nuclei of hBMSCs (scale bar = 20 μm). Quantitative analysis of pYAP/tYAP ratio of hBMSCs on different substrates (B) and on hPC substrate with different treatments (C) (*n* = 3; * *p* < 0.05). (D) Immunohistochemistry graph showed the disruption of YAP nuclear import by Cyto D or Pitstop-2 (scale bar = 50 μm).

As mechanosensors and mechanotransducers, Yes-associated protein (YAP) plays an important role in mediating cellular mechanosensing process.^68^ Increasing of cellular contractility could enhance YAP dephosphorylation and nuclear translocation, and promote cell proliferation as well as stem cell osteogenic differentiation.^12,69,70^ Here, it was found that the cells on hPC showed a higher level of YAP activity, as evidenced by an enhanced YAP nuclear localization (Figure 4A) and a lower pYAP/tYAP ratio (Figure 4B), in comparison to the cells on sPC and mPC. The treatment with inhibitors (RGD, Bleb, and Cyto D) to block the mechanotransduction pathways significantly increased the pYAP/tYAP ratio of hBMSCs on hPC (Figure 4C), which confirmed the effect of the cell contractile force on YAP activity.

In addition, YAP activity was regulated by nuclear component proteins; as demonstrated by siRNA transfection experiments, interference of the nucleoskeleton and LINC complex proteins improved the pYAP/tYAP ratio (Figure 4C). Interfering force transmission with Cyto D and disrupting the nuclear pore complex (NPC) permeability barrier with Pitstop-2 retarded the YAP nuclear accumulation on hPC (Figure 4D). This finding is consistent with a previous study, in which the mechanical force applied to the nuclear membrane resulted in an enhancement of NPC permeability, facilitating the import of mechanotransduction signaling molecules.^19^

### hPC Substrate Microstructure Modulates Histone Modification

**Figure 5:**
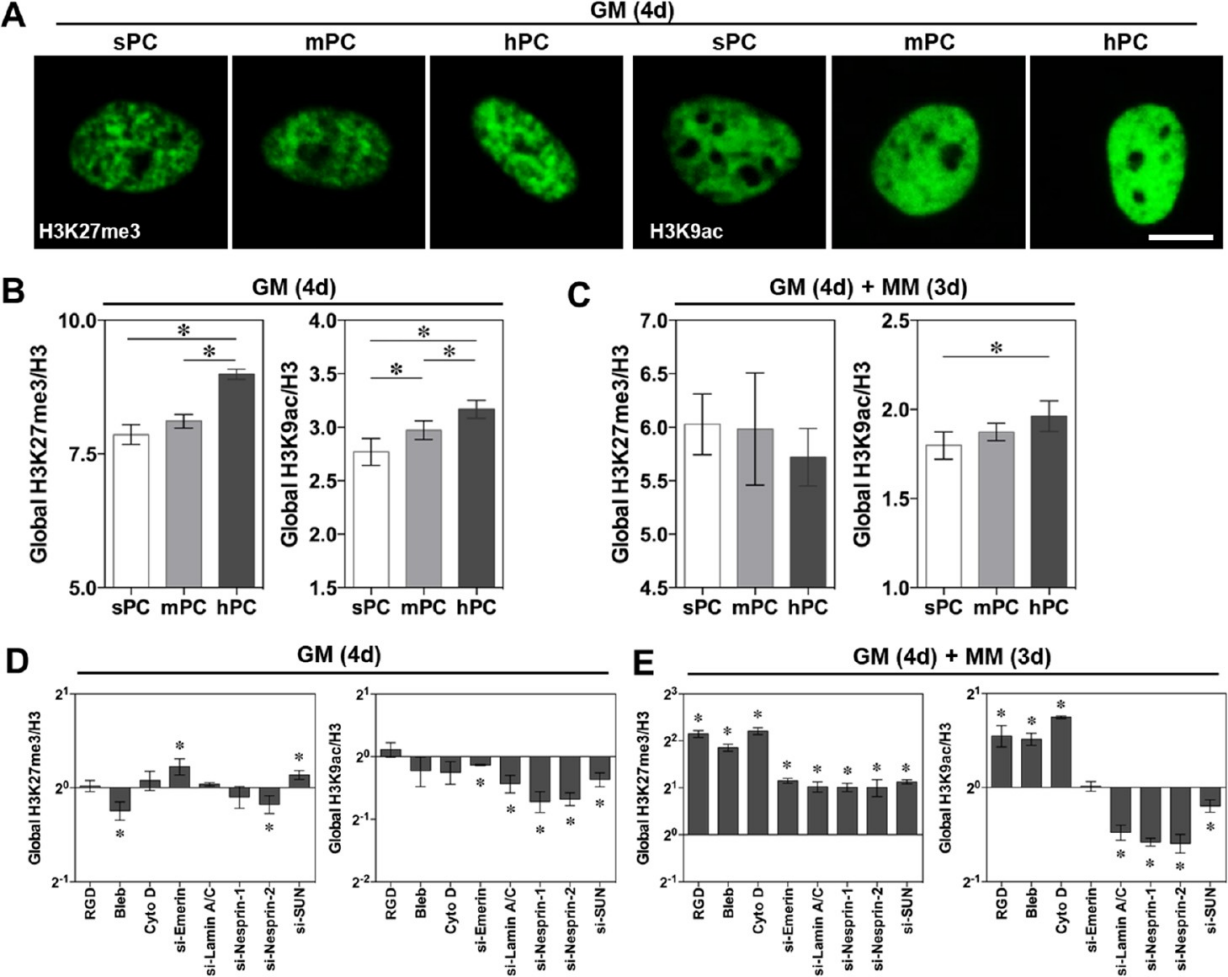
Substrate topographical cues regulate global histone modification. The cells were either cultured in GM for 4 days (GM (4d)) or with additional 3 days of culture in MM (GM (4d) + MM (3d)). (A) Representative H3K27me3 and H3K9ac immunofluorescence staining images of hBMSCs on different substrates (scale bar = 10 μm). (B,C) Quantification of H3K27me3/H3 and H3K9ac/H3 ratio of MFI using flow cytometry. The MFI was calculated with Flowjo software (*n* ≥ 4; * *p* < 0.05). (D,E) Quantification of globe H3K27me3 and H3K9ac in hBMSCs on hPC under different inhibiting conditions (*n* = 5; * *p* < 0.05).

The histone H3K27 trimethylation (H3K27me3) and H3K9 acetylation (H3K9ac) present the epigenetic control points of MSC differentiation,^31^ and their modification levels are directly related to the chromatin compaction degree.^71^ Here, we examined the global H3K27me3 and H3K9ac expression levels of cells cultured on different substrates. When cultured in GM, cells on hPC presented a stronger H3K27me3 and H3K9ac fluorescence intensity in the nuclei, compared to the cells on sPC and mPC (Figure 5A). The cells on hPC had a significantly higher H3K27me3/H3 and H3K9ac/H3 ratio than on mPC and sPC according to the quantitative analysis (Figure 5B), suggesting a promoted stemness of hBMSCs on hPC.^72,73^ However, when the medium was changed to MM for an additional 3 days of culture, the difference of H3K27me3/H3 ratio in different groups was diminished (Figure 5C). This might be explained by the decrease of H3K27me3 during the process of MSC osteogenic differentiation, which plays a critical role to regulate the expression of osteogenic genes.^74^ Notably, the cells on hPC retained the highest H3K9ac/H3 ratio in MM (Figure 5C). This observation was supported by the enhanced nuclear export of the phosphorylated histone deacetylase 1 (pHDAC1) on hPC (Figure S7A,B), which interferes with histone acetylation when it presents intranuclearly.

Previous studies have shown that the cell-extrinsic force propagated directly to the nuclei could increase the Lamin A/C expression and cell nuclei stiffness, resulted in the loosening of chromatin structure by histone modification,^75^ as well as modified genes expression patterns and cell fate.^76^ In order to identify the functions of mechanotransduction proteins at the epigenetic level, we next examined the histone modification on hPC substrate using inhibitors and siRNAs. Notably, the cell culture medium showed a dramatic influence on the global histone modification (Figure 5D,E). In MM, blockage of integrin, myosin, and F-actin significantly increased both the global H3K27me3/H3 and H3K9ac/H3 ratios. However, blockage of nuclear proteins resulted in the enhanced H3K27me3 level and reduced H3K9ac level. These results confirmed the previous reports that osteogenesis of MSCs was accompanied by loss of H3K27me3 and enrichment of H3K9ac at the global level^74,77^ and identified the functions of the examined cytosolic and nuclear proteins in regulating MSC differentiation. In addition, we found that siRNA interference of nuclear proteins led to the nuclear retardation of pHDAC1 (Figure S8A, B), which explained the resulting decrease of global H3K9ac.

**Figure 6:**
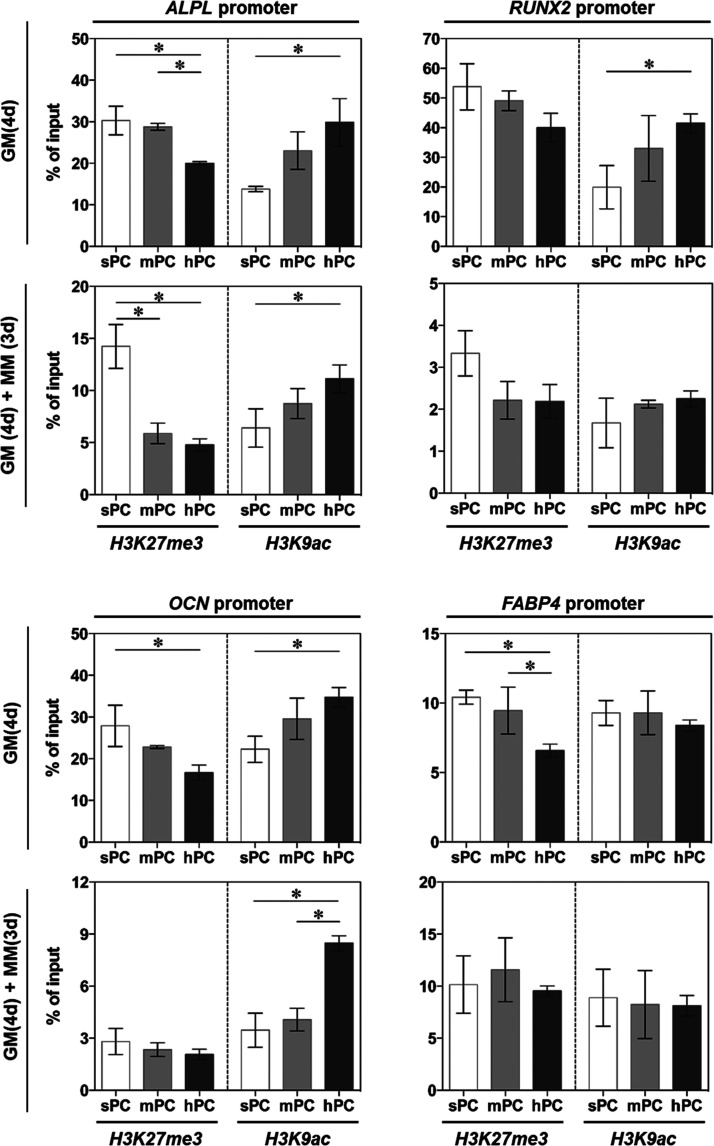
Substrate topographical cues influence the histone modification on the promoters of differentiation genes. The cells were either cultured in GM for 4 days (GM (4d)) or with additional 3 days of culture in MM (GM (4d) + MM (3d)). ChIP-PCR was performed to examine the H3K27me3 and H3K9ac on the promoters of osteogenesis and adipogenesis genes (*n* = 3; * *p* < 0.05).

As a negative regulator, the enrichment of H3K27me3 on specific genes is directly linked to downregulated gene expression.^78^ In contrast, H3K9ac plays an important role in regulating the “switch on” of gene transcription, which is crucial for MSCs osteogenic differentiation.^79^ Here, H3K27me3 and H3K9ac on the transcriptional control regions of differentiation related genes were examined using ChIP-PCR. The hBMSCs cultured on hPC in both GM and MM showed significantly lower H3K27me3 but higher H3K9ac on the promoters of osteogenesis genes (alkaline phosphatase (*ALPL*), RUNX family transcription factor 2 (*RUNX2*) and *OCN*), as compared to the cells on other substrates. However, the histone modification at the promoters of adipogenesis gene *FABP-4* was marginally influenced by the substrate microstructure, with the exception of decreased H3K27me3 on hPC in GM (Figure 6).

### Histone Modification Regulated by YAP and Lamin A/C

YAP and transcriptional coactivator with PDZ binding domain (TAZ) can interact with various histone-modifying enzymes to regulate histone methylation and acetylation, which in turn affect gene expression. For example, YAP and TAZ transcriptionally regulate the histone methyltransferase enhancer of zeste homologue 2 (EZH2),^80^ which plays a role of gene silencing by catalyzing H3K27 trimethylation.^81^ The nuclear transportation and activation of YAP/TAZ can cause histone acetylation by the related lysine acetyltransferases CBP and p300.^82^

Despite the fact that HIPPO pathway or YAP mediated alterations in chromatin accessibility have received great attention in recent studies,^83,84^ it remains unclear whether active YAP can facilitate histone modifications at specific differentiation gene promoters in response to topographical or mechanical cues.^85−87^

**Figure 7:**
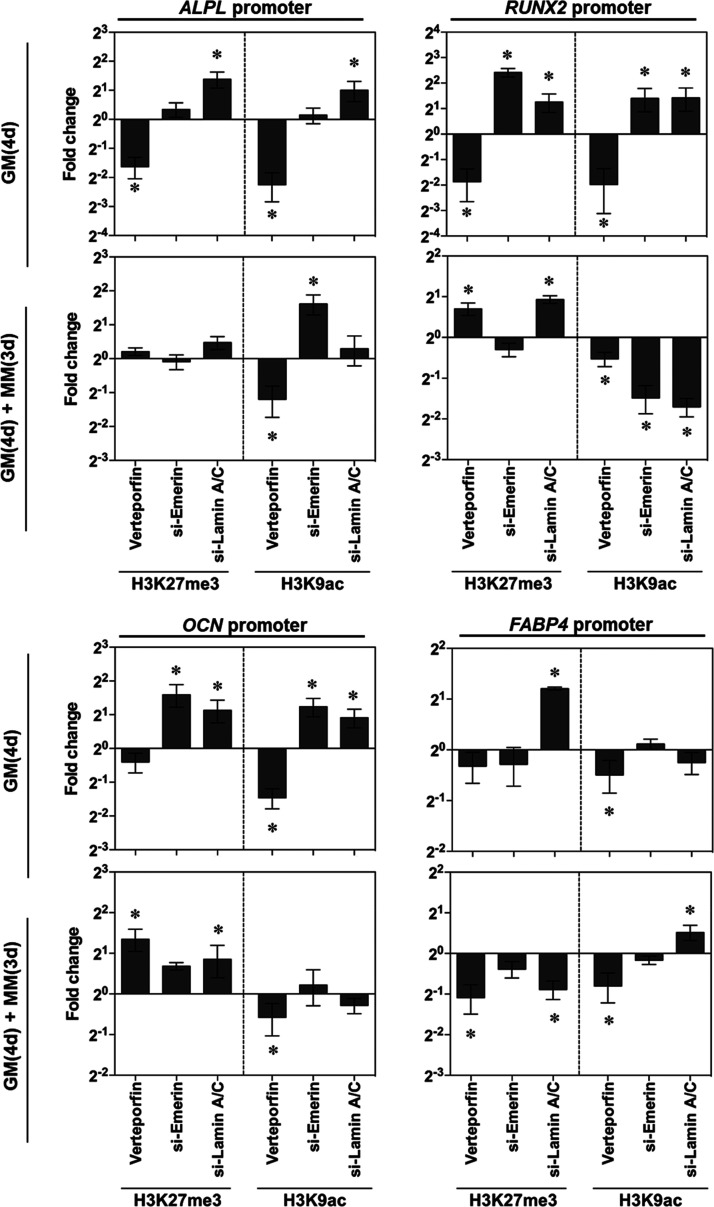
Regulation of YAP activity and nucleoskeleton on histone modification at the promoters of osteogenesis genes (*ALPL, RUNX2, OCN*) and adipogenesis gene *FABP-4*. The cells were either cultured in GM for 4 days (GM (4d)) or with additional 3 days of culture in MM (GM (4d) + MM (3d)) (*n* = 3; * *p* < 0.05).

Here, we hypothesize that YAP, together with other nuclear proteins, may regulate histone modification and expression of differentiation related genes in the mechanotransduction processes. Therefore, we inhibited YAP, Emerin, and Lamin A/C to evaluate the regulation of intracellular and nuclear force transmission on histone modification at differentiation gene loci (Figure 7). In GM, YAP inhibition decreased H3K27me3 and H3K9ac for all examined genes, while Lamin A/C inhibition increased H3K27me3 and H3K9ac for osteogenesis genes. Blockage of Emerin led to the increase of H3K27me3 and H3K9ac on *RUNX2* and *OCN*. When the differentiation process was initiated by culturing in MM, the effects of YAP inhibition on H3K9ac was similar as in GM. Notably, YAP inhibition significantly enhanced H3K27me3 on *RUNX2* and *OCN*, but suppressed H3K27me3 on adipogenesis gene *FABP-4*, suggesting its role for promoting osteogenesis. The effect of Emerin on histone modification was dependent on the genes, as evidenced by H3K9ac enhancement on *ALPL* and reduction on *RUNX2* upon Emerin interference. The blockage of Lamin A/C led to the H3K27me3 increase on *RUNX2* and *OCN* and decrease on *FABP-4* promoter, as well as the H3K9ac decrease on *RUNX2* and increase on *FABP-4*, suggesting that Lamin A/C promoted osteogenesis and suppressed adipogenesis in MM. These results suggested that the hPC topographical cues could affect hBMSC differentiation at the epigenetic level by regulating histone modification via LaminA/C and YAP activation.

### Mechanotransduction Signaling Cascades in hBMSC Differentiation

**Figure 8:**
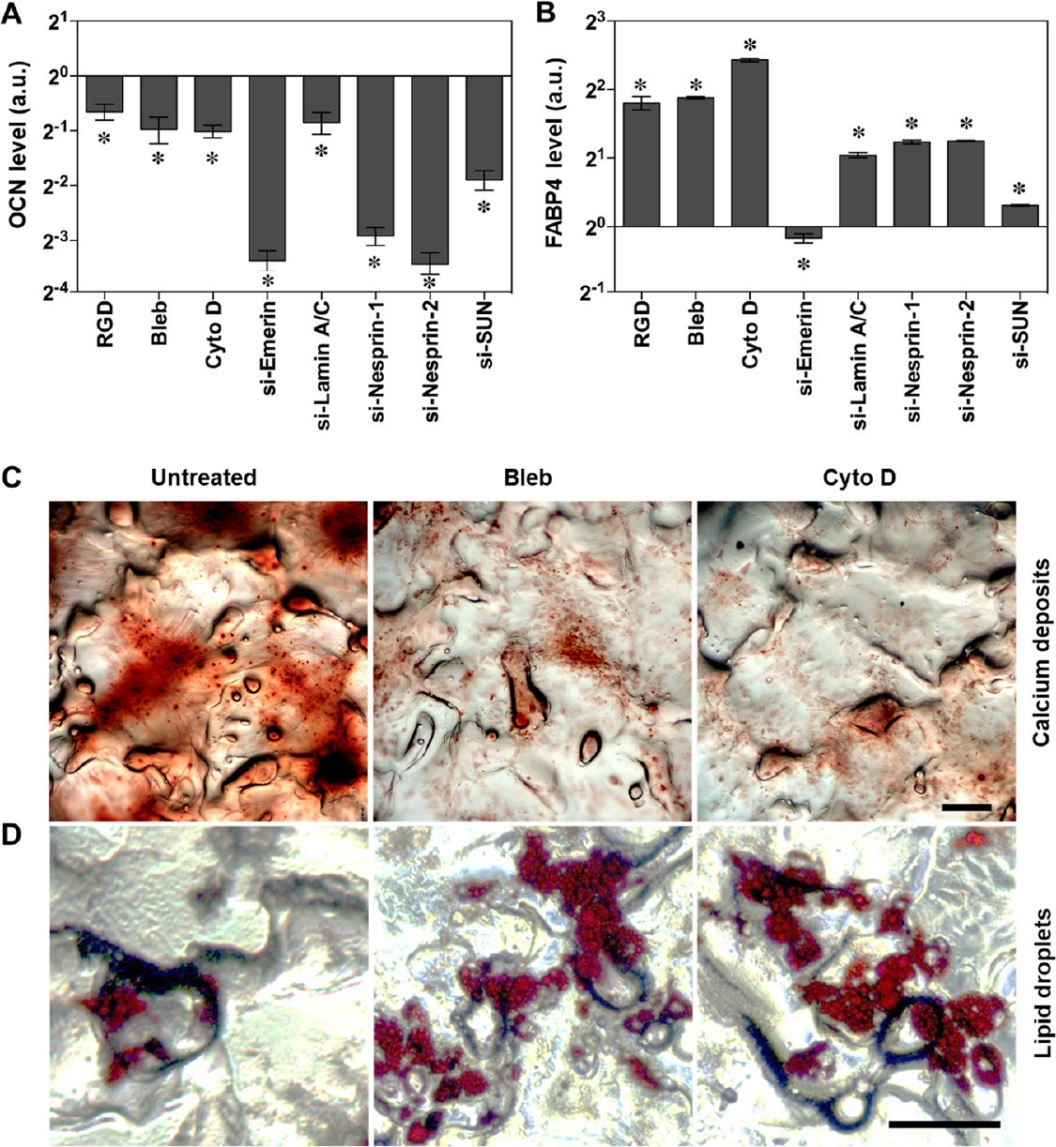
Mechanical force mediated hBMSC differentiation toward osteolineage. The cells were first cultured in GM for 4 days to reach a confluence of 80%; then the medium was changed to MM for 14 additional days of culture. The cells were treated with inhibitors or siRNAs, and their differentiation was examined via quantification of OCN (A) and FABP-4 (B) (*n* = 4; * *p* < 0.05) and staining of calcium deposits (C) and lipid droplets (D) (Scale bar = 200 μm).

At the end, we examined the effects of mechanical force on differentiation capacity of hBMSCs growing on hPC in MM by inhibiting the components involved in the mechanotransduction cascade. As expected, the expression levels of osteogenesis marker OCN significantly decreased after the inhibition of integrin, myosin, F-action or nuclear envelope proteins (Figure 8A). In contrast, the levels of adipogenesis marker FABP-4 increased after the inhibitions except for a slight decrease induced by Emerin interference (Figure 8B). Consequently, the calcium deposition (Figure 8C) was strongly suppressed, while lipid droplets formation (Figure 8D) was enhanced by disrupting contractile filaments or inhibiting actin polymerization.

**Figure 9:**
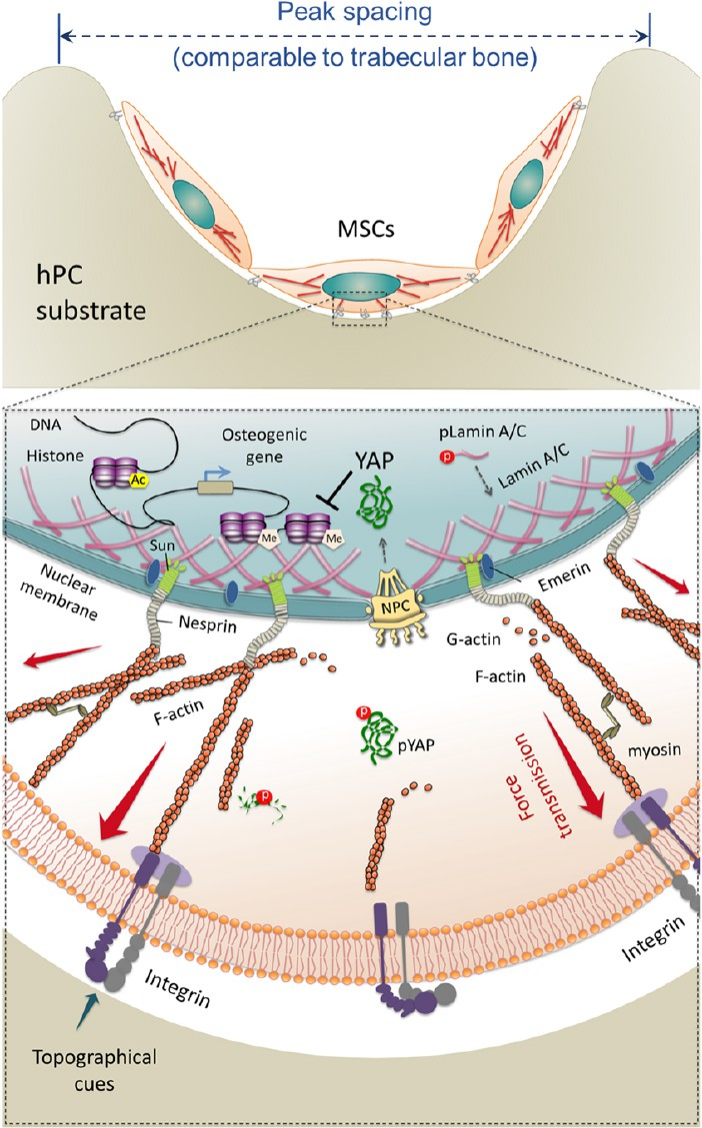
Schematic illustration of intracellular and intranuclear signals triggered by hPC topographical cues, which promote hBMSC osteogenic differentiation.

In summary, these results demonstrate that the hPC substrate with topographical cues mimicking the native trabecular bone could trigger the intracellular and intranuclear biological signals, and enhance the osteogenic differentiation of hBMSCs (Figure 9). After cell attachment, the unique topographical cues presented by hPC such as the peak spacing comparable to that of trabecular bone could be sensed by the cells, and then a series of signal pathways and functional proteins were activated. These include the promoted integrin expression and activation, focal adhesion formation, FAK phosphorylation, and downstream signaling. The mechanical signals transmitted across the cytoplasmic membrane could promote the actin polymerization and myosin activation, which enhanced the cell contractile force. The contractile force induced cell nuclei stretching through the cytoskeleton and the LINC complex, resulting in cell nuclei elongation, laminA/C up-regulation on inner nuclear membrane, and YAP activation. The mechanical force propagated into the nuclei could directly affect the chromatin organization, histone modification, and accessibility of DNA for transcription,^88^ regulating the cells at both epigenetic and genetic levels. As a result, the hBMSCs showed enhanced osteogenic differentiation on hPC substrate. This study focused on the mechanism of substrate topographical cues regulating stem cell differentiation, which pointed to a gateway to control MSC function critical for their therapeutic potential by specifying cell–material interactions. Our results suggest that fine-tuning of the surface microstructure might be an effective and safe approach to improve the therapeutic functions of stem cells. For example, electric discharge machining could be used to modify the surface of bone implants to create a peak spacing similar to the pore size of trabecular bone.^89^ Such an implant preloaded with MSCs might accelerate the bone regeneration process. This study provides design criteria for cell culture substrate and implant surface.

## Conclusions

In this study, inspired by the structure of trabecular bone, we designed the 2.5D substrate with an average peak spacing comparable to the pore size of trabecular bone. Such a substrate could enhance hBMSCs adhesion and cytoskeleton organization and promote cytoskeleton tension. The increased cytoskeleton tension would lead to cell nuclear deformation and histone modification, which further regulated gene expression and directed hBMSC differentiation lineage. These findings fill a critical gap in our basic understanding of cell–substrate interaction and highlight the substrate topographical cues as an important design factor for regulating stem cells in bone repair and regeneration. Such information is critical to future improvement in implants as it points out a new direction to design and develop implant devices. The mechanism study identified the functions of different components including the transmembrane, intracellular, and nuclear proteins at the epigenetic level. Especially, we demonstrated the role of YAP and Lamin A/C for histone modification at the differentiation gene loci in the mechanotransduction process. These results present the perspective for using materials together with biochemical factors targeting to YAP and Lamin A/C, to maximize the potential of stem cell in application.

## Experimental Section

### Polycarbonate Inserts for Cell Culture

Polycarbonate (PC, trade name Makrolon 2805, Bayer, Germany) inserts with the suitable size to put into the standard 24-well tissue culture plates were fabricated via injection molding,^90^ using three modules with different surface structures (a module with a polished contact surface and two modules with microstructured surfaces according to the norm DIN 16747:1981–05, M30 and M45). The prepared inserts were packaged and sterilized by gas sterilization (gas phase: 10% (v/v) ethylene oxide, 54 °C, 65% relative humidity, 1.7 bar, 3 h of gas exposure time and 21 h of aeration phase).

The substrates were characterized at both micro- and nanoscale with respect to their arithmetic average roughness (Ra), root-mean-squared roughness (Rq), and mean spacing between peaks (Sm). The microstructure on the inset bottom was examined using an optical profilometer (MicoProf 200), and the results were analyzed using the software AQUIRE (ver. 1.21) and MARK III (ver. 3.9) according to the previously reported method.^91^ Surface nanotopography was measured using an atomic force microscope (AFM, MFP-3D, Asylum Research, Santa Barbara, CA, USA). A silicon cantilever (OMCL-AC160TS-R3, Olympus, Tokyo, Japan) with a spring constant of 9 N/m was used for AC-mode scanning. For each sample, an area of 2 × 2 μm^2^ at six different locations was scanned in the dry state at ambient temperature with a scan rate of 0.5 Hz. The results were analyzed using Igor Pro 6.22A software.

### Cell Culture

hBMSCs were purchased from Merk Millipore (SCC034, Merk Millipore, Darmstadt, Germany). MesenPRO RS growth medium (GM) (ThermoFisher Scientific, Waltham, USA) was used for cell maintenance. 2 × 10^4^/cm^2^ cells were seeded and cultured in a humidified atmosphere containing 5% (v/v) CO_2_, and the medium was changed every 2 days. For experiments in conditions to induce differentiation, 2 × 10^4^/cm^2^ cells were seeded and precultured in GM for 4 days to reach 80% confluence and subsequently replaced with the mixed induction media (MM) (osteogenic induction medium: adipogenic induction medium = 1:1 (*v:v*)) for additional 3, 14, or 21 days to induce the cell differentiation using common media supplements.^58^ The StemPro Osteogenesis differentiation kit and StemPro Adipogenesis differentiation kit (ThermoFisher Scientific, Waltham, USA) were applied to promote osteogenic and adipogenic differentiation, respectively.

### Collagen Matrix-Based Contractility Assay

The contractility of hBMSCs was evaluated using a collagen matrix-based cell contraction assay kit (Cell Biolabs, Inc., California, USA). Single cell suspension of day 4 hBMSCs (4 × 10^3^ cells/μL) in GM was collected and mixed with collagen solution (1:4 v/v). A 250 μL mixture of cell–collagen was added into each well of a 48-well plate and incubated at 37 °C for 1 h; then 400 μL GM was added to each well and incubated for 2 days. The cell–collagen gels were then released from culture plates. The shape of the gels were recorded by camera at indicated time points, and the gel size was measured via ImageJ software (National Institutes of Health, USA).

### Flow Cytometry

Cells were fixed with 4% (w/v) paraformaldehyde, permeabilized with 0.1% (v/v) Triton X-100 (Sigma-Aldrich Chemie GmbH, Taufkirchen, Germany), and stained with Alexa Fluor 488 conjugated mouse anti-active Integrin β1 (Sigma-Aldrich Chemie GmbH, Taufkirchen, Germany), Alexa Fluor 488 conjugated rabbit anti-H3K27me3, Alexa Fluor 647 conjugated rabbit anti-H3K9ac, Pacific Blue conjugated mouse anti-histone H3 antibodies (Cell signaling technologies, Danvers, USA), and mouse anti-Lamin A/C primary antibody for overnight at 4 °C. The sample for Lamin A/C assay was incubated with anti-mouse IgG (H+L)-Alexa Fluor 633 (Invitrogen, California, USA) for another 30 min at room temperature. The data were recorded by MACSQuant flow cytometer (Miltenyi Biotec, Bergisch Gladbach, Germany) and analyzed using “Flowjo” software (Tree Star Inc., Ashland, OR, USA).

### Western Blotting

Cells were lysed by the RIPA lysis and extraction buffer (Thermo Fisch scientific, Waltham, Massachusetts, USA) containing a mixture of 1× halt protease and phosphatase inhibitor cocktail (Thermo Fisher Scientific, Waltham, Massachusetts, USA) on ice for 10 min. The total protein concentration in the supernatant was measured using a BCA Protein Assay Kit (Thermo Fisher Scientific, Bonn, Germany). For quantification of G-actin/F-actin ratio, the G-actin/F-actin assay kit (Cytoskeleton Inc., Denver, USA) was used, hBMSCs were lysed by F-actin stabilization buffer. Then the lysate was centrifuged by ultracentrifuge (Sorvall/Thermo Scienfic, Massachusetts, USA) at 100 000 × g, at 37 °C for 1 h. The F-actin (pellet) and G-actin (supernatant) were separated. The pellets were subsequently resuspended in the ice-cold depolymerizing buffer for analysis. Then loading buffer (Bio-Rad, München, Germany) was added to F-actin, G-actin sample, and all cell lysates and boiled at 95 °C for 5 min. Then equal amounts of samples were loaded to a 10% SDS-PAGE gel for electrophoresis at 120 V for 60 min, and the protein was transferred onto the nitrocellulose membrane (Merck Millipore, Darmstadt, Germany) at a constant current of 220 mA for 70 min. The membrane was blocked with Odyssey Blocking Buffer (LI-COR Biosciences, Lincoln, NE, USA) and stained with anti-Lamin A/C, anti-pLamin A/C, anti-N-cadherin, and anti-GAPDH antibodies (Cell Signaling Technologies, Danvers, USA) overnight at 4 °C. The secondary antibody IRDye 800CW (LI-COR Biosciences, Lincoln, NE, USA) was then added and the protein bands were detected using an Odyssey Infrared Imaging System (LI-COR Biosciences, Lincoln, NE, USA). The protein level was quantified by analyzing the intensity of bands with ImageJ software (National Institutes of Health).

### Enzyme-Linked Immunosorbent Assay (ELISA)

The pFAK, tFAK, pMLC, pYAP, and tYAP levels of hBMSCs were measured using pFAK, tFAK (Thermo Fisher Scientific, Bonn, Germany), pMLC (Mybiosurce, San Diego, USA), pYAP (pSer397), and tYAP ELISA kits (Cell signaling technologies, Danvers, USA), respectively. The expression level of osteocalcin (OCN) and fatty acid binding protein 4 (FABP-4) in differentiated hBMSCs (day 14 or day 21 in MM) were quantified by Human OCN-ELISA kit (Invitrogen, California, USA) and Human FABP-4 ELISA kit (Abcam, Cambridge, UK). The results were normalized by the total protein amount of the cell extraction.

### ChIP-PCR Analysis

According to the manufacturer’s protocol of SimpleChIP Enzymatic Chromatin IP Kit (Cell signaling technologies, Danvers, USA), the chromatin immunoprecipitation was performed by using rabbit anti-H3K27me3 and rabbit anti-H3K9ac antibodies (Cell signaling technologies, Danvers, USA). The precipitated DNA was purified and eluted by incubating with RNase and proteinase K overnight. The DNA was amplified with RT2 SYBR Green ROX qPCR Mastermix (Qiagen, Hilden, Germany) using a StepOnePlus System (Thermo Fisher Scientific, Bonn, Germany). The sequences of primers (Thermo Fisher Scientific, Bonn, Germany) are listed in Supplementary Table S2. The enrichment was calculated relative to input DNA data and expressed as percent of Input = 2% × 2^(CT 2% input sample-CT ChIP sample)^.

### Statistical Analysis

The number of replications for quantitative experiments was equal to or larger than three as indicated respectively in the figure legends. Unless indicated otherwise, the data were expressed as arithmetic mean ± standard deviation. The significance of the difference between two groups was determined using a two-tailed independent sample *t* test. Differences among three or more independent groups were analyzed using one-way ANOVA followed by Tukey’s test (multiple comparisons) or Dunnett’s test (comparison of inhibitor or siRNA treated group with the corresponding control group). A *p* value less than 0.05 was considered to be statistically significant.

## Supporting Information Available

Supporting methods of cell staining, inhibition, siRNA transfection; supporting tables of characterization of surface micro- and nanoscale topography, sequences of ChIP-PCR primers; supporting figures of surface profiles of the sPC, mPC and hPC, N-cadherin expression, hBMSC differentiation on different surfaces, cell contractility assay, distribution of cells on hPC, knockdown efficiency of siRNA targeting Lamin A/C, pHDAC1 intracellular distribution in response to topographical cues (PDF)
