# Supplementary material for: Histone Modification of Osteogenesis Related Genes Triggered by Substrate Topography Promotes Human Mesenchymal Stem Cell Differentiation
Source: ACS Appl Mater Interfaces. 2023 Jun 13;15(25):29752–66. doi: 10.1021/acsami.3c01481 (PMC10316329; doi:10.1021/acsami.3c01481)
Supplement: Supplementary file 1 — am3c01481_si_001.pdf [file am3c01481_si_001.pdf]

Supporting Information

Histone Modification of Osteogenesis Related Genes Triggered by Substrate Topography Promotes Human Mesenchymal Stem Cell Differentiation

*Xun Xu [1 ‡], Weiwei Wang [1 ‡], Jie Zou [1, 2 ‡], Karl Kratz [1, 3 #], Zijun Deng [1, 2], Andreas Lendlein [1, 2, 3, 4] * and Nan Ma [1, 2, 3] **

[1] Institute of Active Polymers and Berlin-Brandenburg Centre for Regenerative Therapies, Helmholtz-Zentrum Hereon, Teltow, Germany

[2] Institute of Chemistry and Biochemistry, Free University of Berlin, Berlin, Germany

[3] Helmholtz Virtual Institute − Multifunctional Biomaterials for Medicine, Teltow and Berlin, Germany

[4] Institute of Chemistry, University of Potsdam, Potsdam, Germany

Corresponding Author
* To whom correspondence should be addressed:
Prof. Dr. Andreas Lendlein, Prof. Dr. Nan Ma
Email: lendlein@uni-potsdam.de, nan.ma@hereon.de

This PDF file includes:

Supporting methods
- **Method S1.** Cell staining
- **Method S2.** Inhibition experiment
- **Method S3.** siRNA transfection

Supporting tables
- **Table S1.** Characterization of surface micro- and nano-scale topography via optical profilometer and AFM
- **Table S2.** Sequences of ChIP-PCR primers

Supporting figures
- **Figure S1.** Surface profiles of the sPC, mPC and hPC inserts
- **Figure S2.** N-cadherin expression in hBMSCs
- **Figure S3.** hBMSC differentiation on different surfaces
- **Figure S4.** Cell contractility assay
- **Figure S5.** Distribution of cells on hPC substrates
- **Figure S6.** Knockdown efficiency of siRNA targeting Lamin A/C
- **Figure S7.** pHDAC1 distribution in hBMSCs growing on different substrates
- **Figure S8.** pHDAC1 distribution in hBMSCs growing on hPC with siRNA interferences

Supporting methods

Method S1. Cell staining

All hBMSC samples were fixed with 4% (w/v) paraformaldehyde (Sigma–Aldrich, St. Louis, MO USA), permeabilized with 0.1% (v/v) Triton X-100 (Sigma–Aldrich, St. Louis, MO, USA), and blocked with 3% (w/v) BSA (Sigma–Aldrich, St. Louis, MO, USA). Samples were immunostained by mouse anti-vinculin (Life Technologies, Darmstadt, Germany), rabbit anti-fibronectin (Abcam, Berlin, Germany), mouse anti-lamin A/C, rabbit anti-phospho-lamin A/C (Ser22), rabbit anti-H3K27me3, rabbit anti-H3K9ac (Cell signaling technologies, Danvers, USA) and rabbit anti-phosphor-HDAC1 primary antibodies (Life Technologies, Darmstadt, Germany). Anti-mouse IgG (H+L)-Alexa Fluor® 488, and anti-rabbit IgG (H+L)-Alexa Fluor® 633 were used as secondary antibodies (Invitrogen, California, USA). F-actin was stained with ActinRed™ 555 Ready Probes (Life Technologies, Darmstadt, Germany). Cell nuclei were stained with Hoechst 33342 (Life Technologies, Darmstadt, Germany). Oil Red O (Sigma–Aldrich, St. Louis, MO, USA) staining was used to assess the lipid droplets in cells undergoing adipogenesis. OsteoImage™ Mineralization Assay kit (Lonza, Basel, Switzerland) and Alizarin Red S (Sigma–Aldrich, St. Louis, MO, USA) was used for observation of hydroxyapatite and overall calcium deposition during osteogenesis. Bright-field images were obtained by using inverted light microscopy (Axiovert 40C, Carl Zeiss, Jena Germany) and fluorescence images were captured using a confocal laser scanning microscope (LSM 780, Carl Zeiss, Jena, Germany).

Method S2. Inhibition experiment

Inhibitors were added to cell culture medium (GM or MM) at the following concentrations: RGD (10 μg/ mL), belbbistatin (Bleb) (1 μM), cytochalasin D (Cyto D) (0.2 μM), Verteporfin (5 nM) and Pitstop-2 (15 μM) (Sigma-Aldrich, St. Louis, MO, USA). Vehicle (0.1% (v/v) DMSO; PAN-Biotech GmbH, Aidenbach, Germany) served as a negative control. Verteporfin and Pitstop-2 were applied 48 h before cell harvesting. Medium with the other inhibitors was regularly changed in every 2 days.

Method S3. siRNA transfection

Lamin A/C, Nesprin-1, Nesprin-2 and SUN siRNAs (Life Technologies, Darmstadt, Germany) were transfected into hBMSCs using Lipofectamine®2000 (Thermo Fisher Scientific,

Waltham, USA). Non-targeting siRNA (Life Technologies, Darmstadt, Germany) was transfected as a scrambled control. Briefly, one day before transfection, hBMSCs were seeded into each insert and cultured in 400 µl medium (GM or MM) without antibiotics. Lipofectamine®2000 was diluted (1:50 v/v) in Opti-MEM® I reduced serum medium (Thermo Fisch scientific, Waltham, Massachusetts, USA) and incubated for 15 min at room temperature. 20 pmol siRNA was obtained by diluting in Opti-MEM® I reduced serum medium. Diluted siRNA and Lipofectamine® 2000 (total volume = 111 µl) were mixed and incubated for another 15 min at room temperature. siRNA-Lipofectamine® 2000 complexes were added to each well, and after 2-day incubation the medium was refreshed. Cells or cell lysates were collected at day 4 after transfection for further Western blot and flow cytometry analysis.

Supporting tables

Table S1. Characterization of surface micro- and nano-scale topography via optical profilometer and AFM.

Sample ID	Micro-roughness		Nano-roughness		Mean spacing between peaks (Sm) [µm]
	Ra (µm)	Rq (µm)	Ra (nm)	Rq (nm)	
sPC	0.05 ± 0.01	0.16 ± 0.10	1.29 ± 0.27	1.75 ± 0.38	-
mPC	2.94 ± 0.11	3.85 ± 0.11	3.02 ± 1.22	4.78 ± 1.62	160.3 ± 8.2
hPC	11.30 ± 0.43	16.12 ± 0.77	4.62 ± 2.46	6.01 ± 2.73	279.3 ± 32.3

Table S2. Sequences of ChIP-PCR primers

**Gene**	Forward primer 5'-3'	Reverse primer 5'-3'
ALPL	TCCAGGGATAAAGCAGGTC	TTAGTAAGGCAGGTGCCAAT
RUNX2	AGGCCTTACCACAAGCCTTT	AGAAAGTTTGCACCGCACTT
OCN	CAAATAGCCCTGGCAGATTC	GAGGGCTCTCATGGTGTCTC
FABP-4	GACAGGAGTGTCCCGAAGAG	GTGCACTGAATTTCCCCCTA

Supporting figures

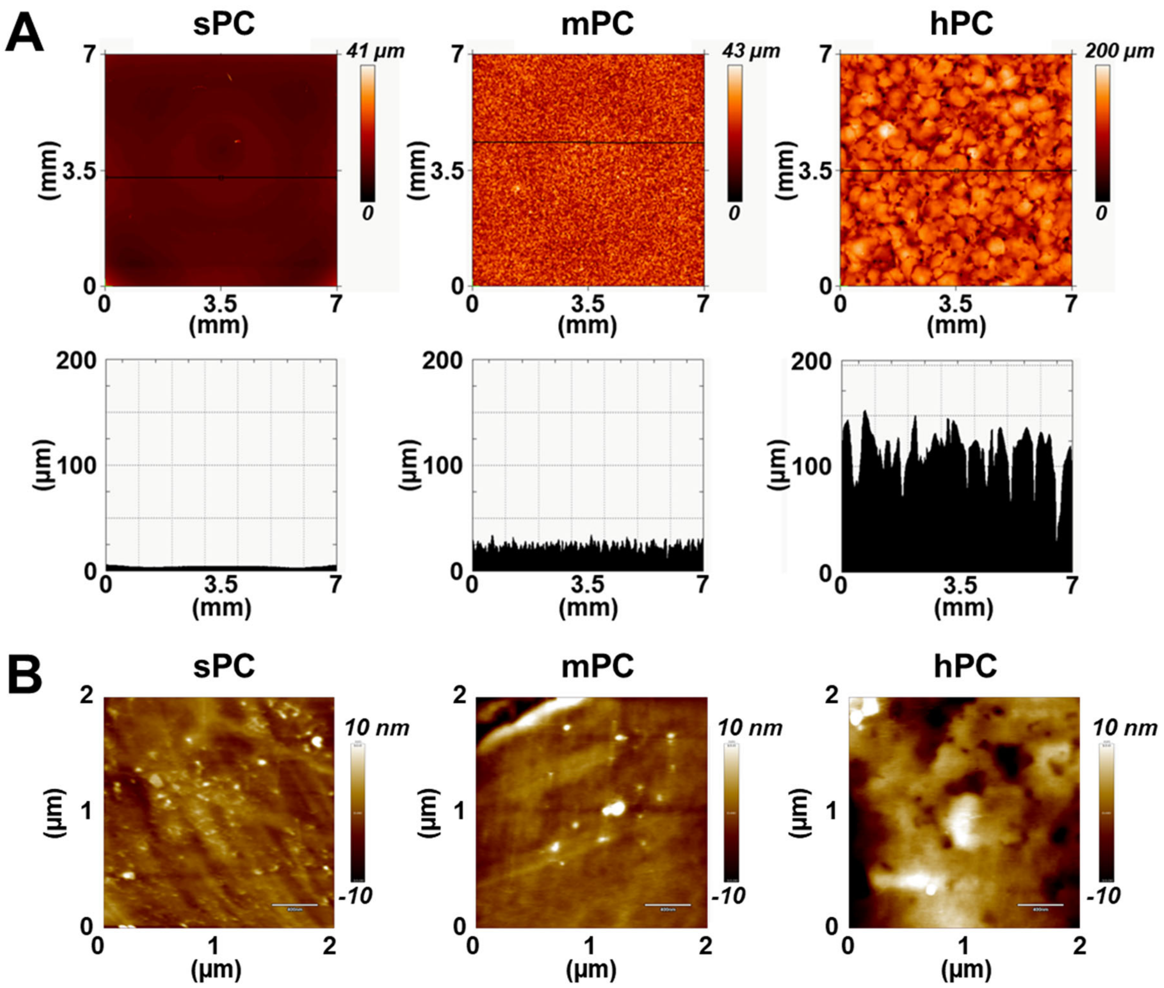

Figure S1. (A) Surface profiles of the sPC, mPC and hPC inserts was determined by optical profilometry at the micro-scale level. (B) Nano-roughness of the substrates was measured using AFM.

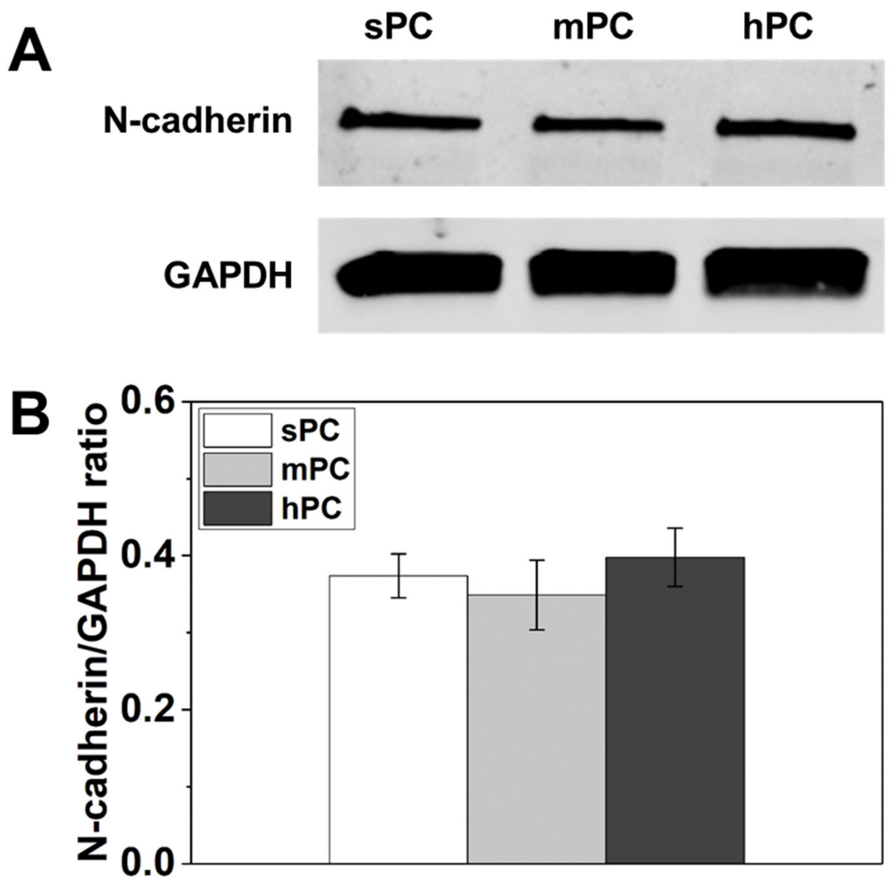

Figure S2. (A) Western blot images of N-cadherin expression in hBMSCs, which were cultured in GM for 4 days. (B) Ratio of N-cadherin/GAPDH via the ImageJ analyzed the grayscale value of bands (n = 4).

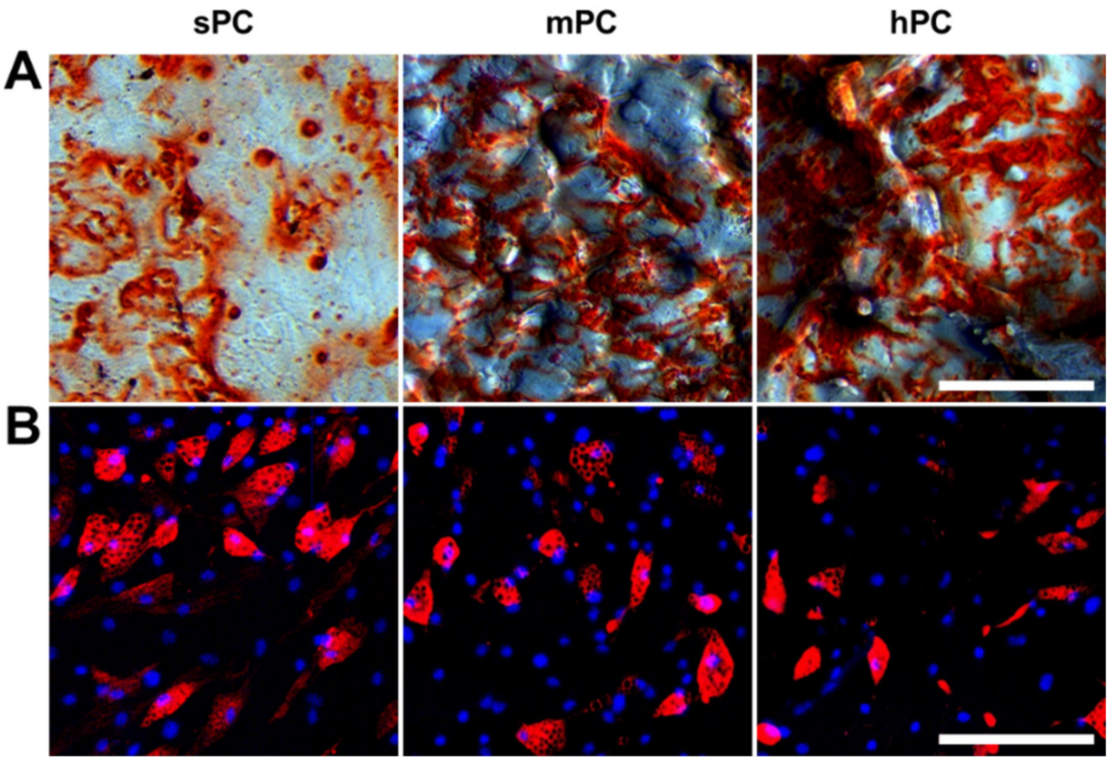

Figure S3. hBMSC differentiation on different surfaces. Representative images of hBMSCs, which were seeded on PC substrates and cultured with osteogenic induction medium and adipogenic induction medium for 21 days, respectively. (A) Images of Aliza red S staining (scale bar = 200 μm); (B) Images of immunofluorescence staining of FABP-4 (red: FABP-4; blue: nuclei; scale bar = 200 μm).

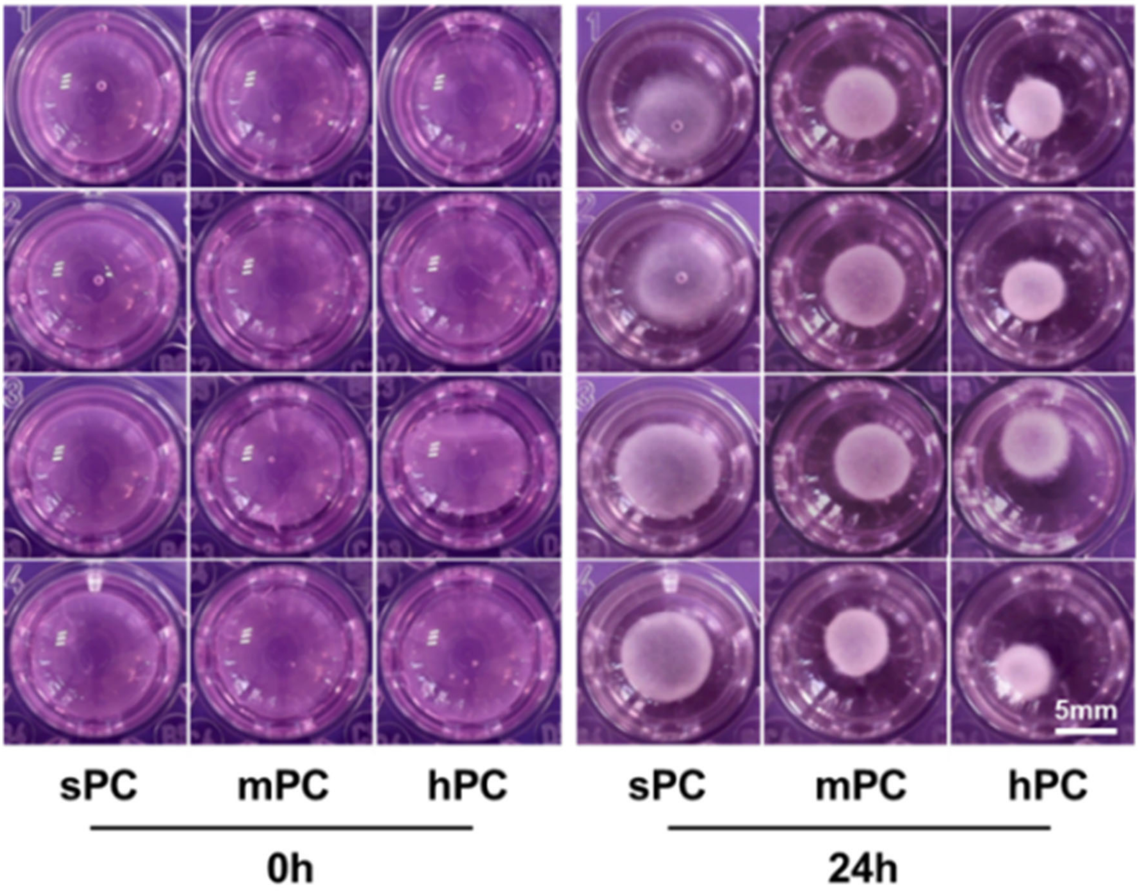

Figure S4. Cell contractility assay. Images of cell mixed collagen gel at 0 h (initial state) and after released at 24 h (scale bar = 5 mm).

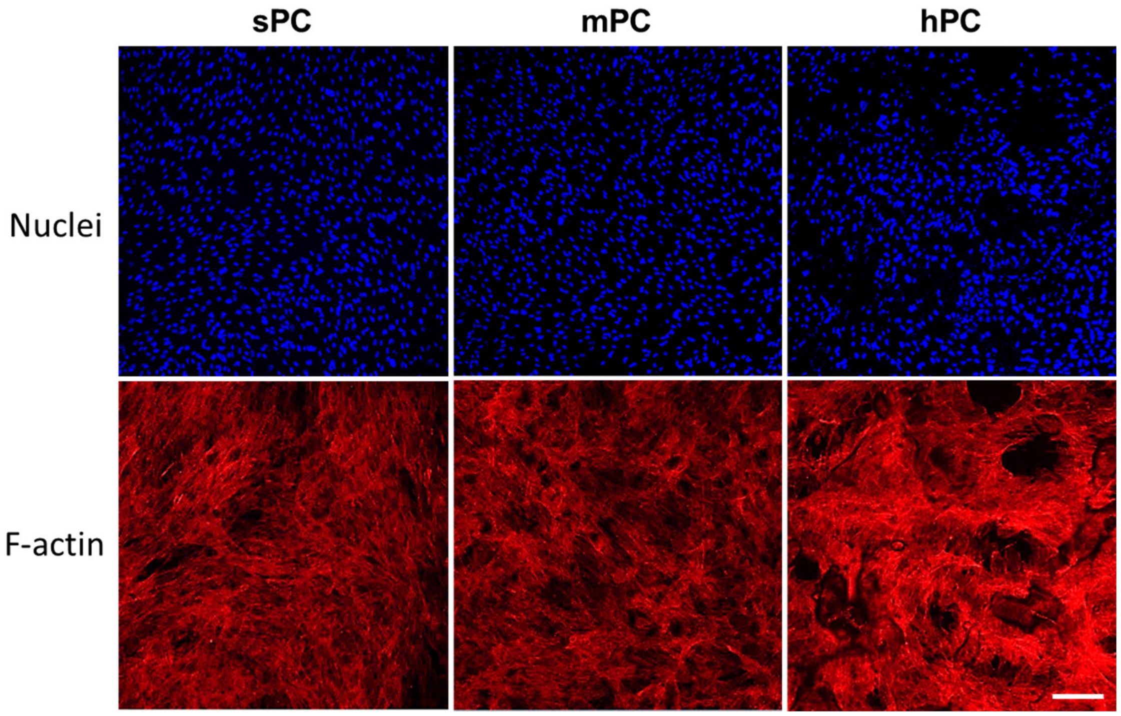

Figure S5. The hPC substrate influenced the distribution of cells cultured at a high density (scale bar = 200 μm).

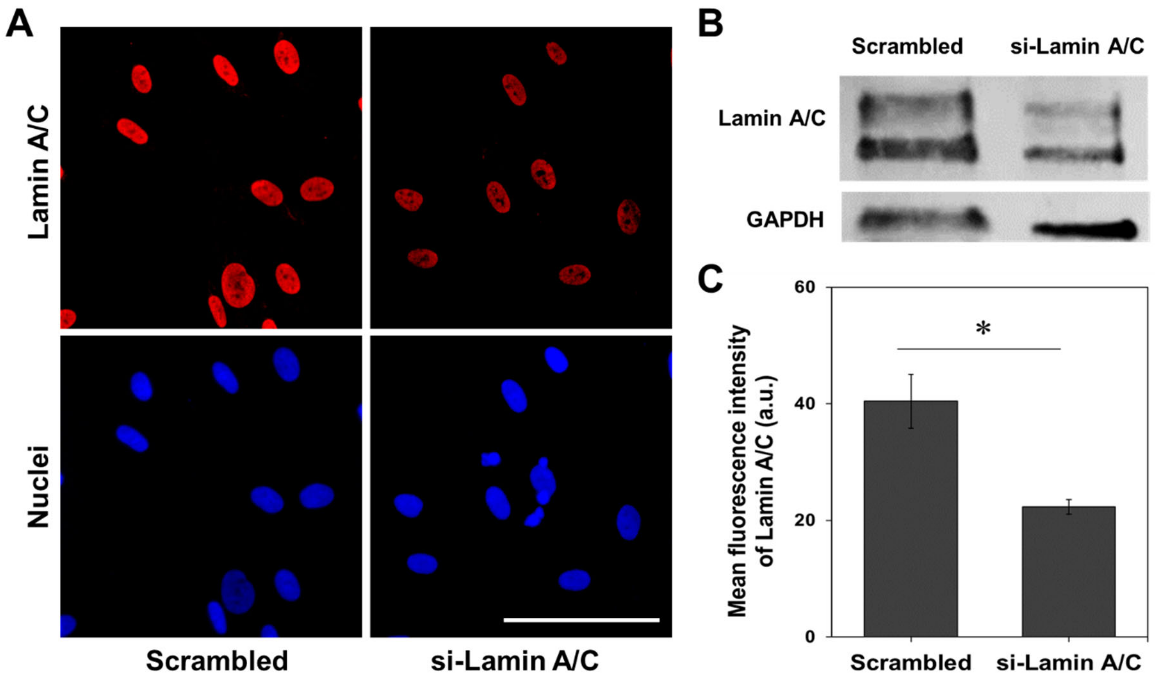

Figure S6. Determination of the siRNA knockdown efficiency targeting to Lamin A/C. Representative images (A), western blotting bands (B) and quantification (C) of Lamin A/C in hBMSCs cultured on hPC treated with scrambled and Lamin A/C specific siRNA (Scale bar = 50 µm ; n_{cells} = 34 (Scrambled), 50 (si-Lamin A/C); * $p < 0.05$).

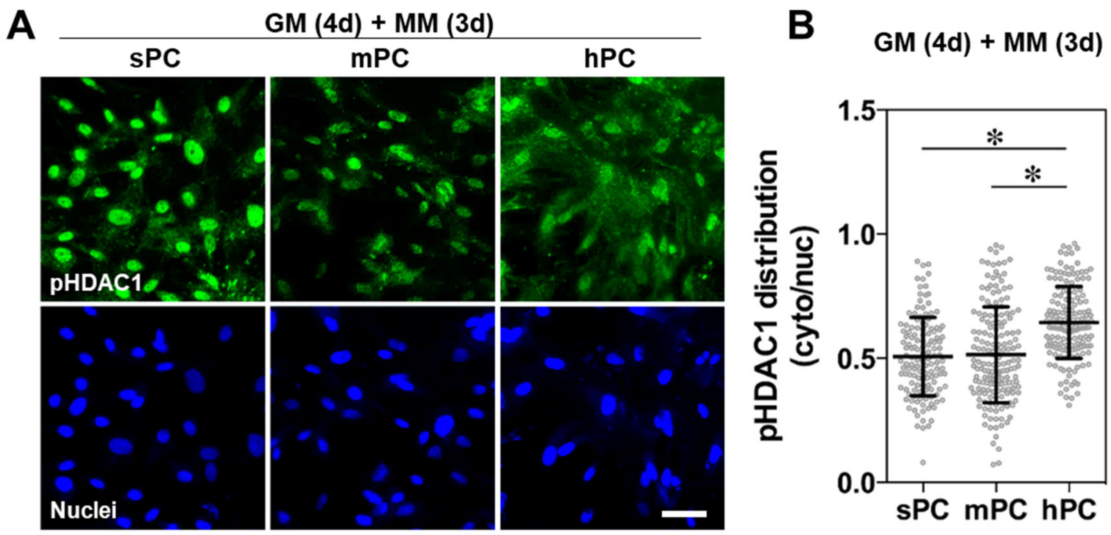

Figure S7. Representative images (A) and quantification (B) of pHDAC1 distribution in hBMSCs growing on different substrates in GM for 4 days and then in MM for additional 3 days (scale bar = 50 µm, n_{cells} = 133 (sPC), 172 (mPC) and 158 (hPC); * $p < 0.05$).

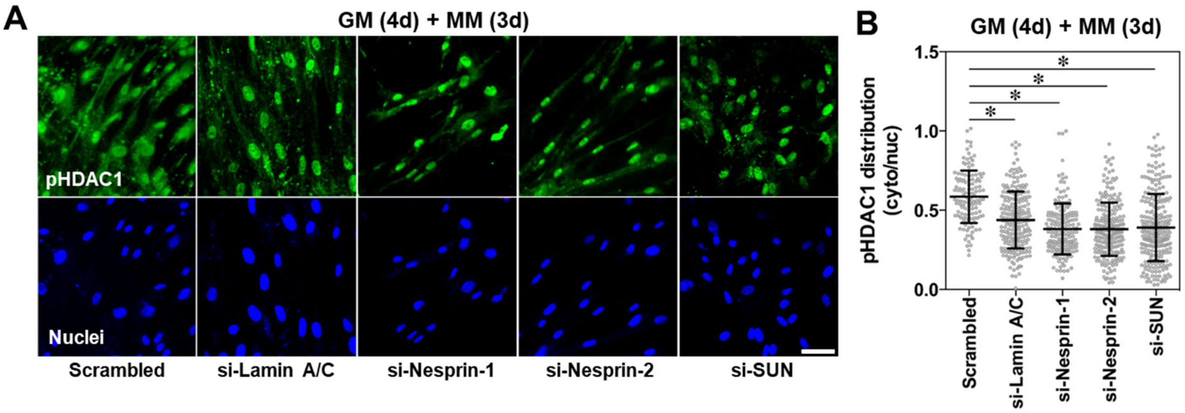

Figure S8. (A) Representative fluorescence images of pHDAC1 and cell nuclei (scale bar = 50 μm). (B) Ratio of fluorescence intensity of the cytoplasmic and nuclear pHDAC1 on hPC in GM for 4 days and then in MM for additional 3 days with different siRNA interferences (n_{cells} = 123 (Scrambled), 217 (si-Lamin A/C), 169 (si-Nesprin-1), 229 (si-Nesprin-2) and 259 (si-SUN); * $p < 0.05$).
